# Visualisation and Analysis of Speech Production with Electropalatography

**DOI:** 10.3390/jimaging5030040

**Published:** 2019-03-15

**Authors:** Jo Verhoeven, Naomi Rachel Miller, Luc Daems, Constantino Carlos Reyes-Aldasoro

**Affiliations:** 1 School of Health Sciences, Division of Language & Communication Science, Phonetics Laboratory, University of London, London EC1V 0HB, UK; johan.verhoeven.1@city.ac.uk or; 2 Department of Linguistics CLIPS, University of Antwerp, 2000 Antwerp, Belgium; 3 Oral and Maxillo-Facial Surgery, ZNA Middelheim General Hospital, B2020 Antwerp, Belgium; 4 School of Mathematics, Computer Science and Engineering, Department of Electrical Engineering, Research Centre in Biomedical Engineering, University of London, London EC1V 0HB, UK

**Keywords:** computed tomography, segmentation, speech production, electropalatography, spectrograms, articulation asymmetry

## Abstract

The process of speech production, i.e., the compression of air in the lungs, the vibration activity of the larynx, and the movement of the articulators, is of great interest in phonetics, phonology, and psychology. One technique by which speech production is analysed is *electropalatography*, in which an artificial palate, moulded to the speaker’s hard palate, is introduced in the mouth. The palate contains a grid of electrodes, which monitor the spatial and temporal pattern of contact between the tongue and the palate during speech production. The output is a time sequence of images, known as palatograms, which show the 2D distribution of electrode activation. This paper describes a series of tools for the visualisation and analysis of palatograms and their associated sound signals. The tools are developed as Matlab^®^ routines and released as an open-source toolbox. The particular focus is the analysis of the amount and direction of left–right asymmetry in tongue–palate contact during the production of different speech sounds. Asymmetry in the articulation of speech, as measured by electropalatography, may be related to the language under consideration, the speaker’s anatomy, irregularities in the palate manufacture, or speaker handedness (i.e., left or right). In addition, a pipeline for the segmentation and analysis of a three-dimensional computed tomography data set of an artificial palate is described and demonstrated. The segmentation procedure provides quantitative information about asymmetry that is due to a combination of speaker anatomy (the shape of the hard palate) and the positioning of the electrodes during manufacture of the artificial palate. The tools provided here should be useful in future studies of electropalatography.

## 1. Introduction

The production of speech sounds consists of the co-ordinated and synchronised movements of an estimated 160 muscles operating the articulators, such as the lips and the tongue, to change the overall shape of the vocal tract (i.e., the mouth–throat channel). These changes in configuration alter the resonant properties of the vocal tract and hence its filter characteristics, which in turn modify, e.g., the periodic sound generated by the vibration of the vocal folds in the larynx [1]. This process enables human beings to produce an immense range of different speech sounds (i.e., vowels and consonants), which are referred to as *phonemes*. In a recent survey of the speech–sound inventories of 1672 languages of the world, it was found that these languages use a total of 2155 different speech sounds [2]. This indicates that the sound production capability of mankind is truly immense considering that languages typically only have 35 speech sounds in their inventory. The production of speech sounds by human beings is studied in the science of (articulatory) phonetics.

In a phonetic perspective, speech sounds are typically described in terms of their manner and place of articulation [3]. Manner of articulation refers to the degree to which the vocal channel is constricted by the articulators. In *plosives*, for instance, the airflow through the vocal tract is fully blocked (e.g., by placing the tip and sides of the tongue against the roof of the mouth), which gives rise to sounds like [p, t, k] and [b, d, g]. In *fricatives*, the tongue is raised and approximates the palate to such a degree that a very small opening remains for the airflow to escape with a clear hissing sound quality. Examples of fricatives are [f, s, v, z]. In *resonants*, the tongue is also raised from its neutral position towards the palate but the gap remains relatively big so that the airstream does not become turbulent and the sound remains without hiss. In English, [j] and [w] are examples of resonants, but this constriction type is also found in most of the English vowels.

Place of articulation refers to the area in the vocal tract where the constriction occurs. In sounds like [t] and [d], for example, the constriction occurs in the alveolar area (i.e., close to the superior alveolar ridge, a small protuberance just behind the upper front teeth). In sounds like [k] and [g], the occlusion occurs in the velar area, with the tongue making contact with the back part of the roof of the mouth. In this perspective, the tongue is a very productive articulator, covering articulations in the dental area (anterior) to those in the pharyngeal area (posterior). In terms of place of articulation, 52% of the speech sounds in languages of the world have an articulation involving the tongue [4].

**Figure 1:**
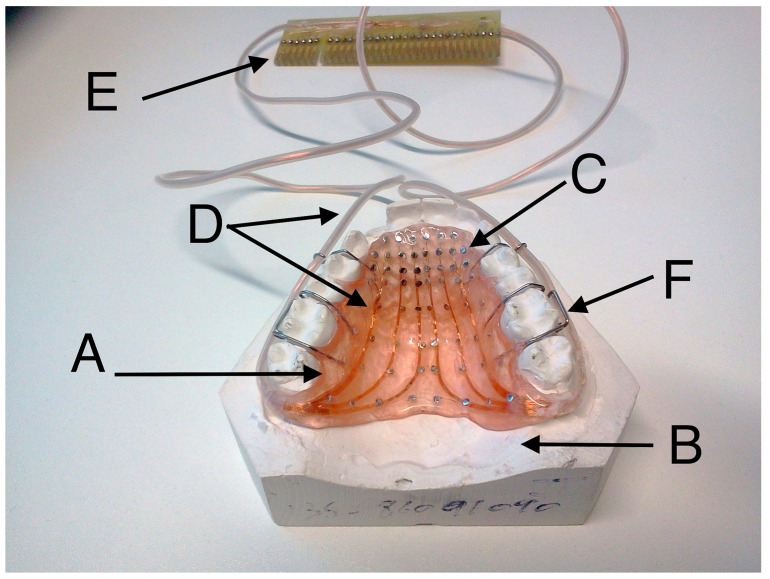
Illustration of a palate used for electropalatography. The palate (A) is crafted over a stone cast of the upper jaw (B) produced by a dentist. The electrodes (C) are positioned manually over the palate and are connected through electrical wires (D) to the interface (E) of the external recording unit. The palate is kept in place with wire clips (F) that are not in contact with the electrical wires or the electrodes.

The place of consonant articulation and the timing of tongue contact with the hard palate can be visualised by means of the technique of electropalatography (EPG) [5,6]. In this instrumental technique, tongue–palate contact is recorded by means of a grid of electrodes placed on the surface of a thin artificial acrylic palate. The artificial palate is custom made to fit against a speaker’s hard palate by obtaining a plaster dental impression of the upper jaw (Figure 1) [7].

**Figure 2:**
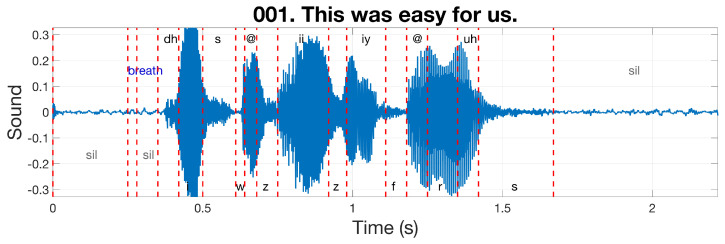
Illustration of an annotated sound wave from the MOCHA (MultiCHannel Articulatory) TIMIT database. The sentence (“This was easy for us”) is displayed as the title of the figure together with the sentence number in the database. The sound wave is displayed as a blue line and the phonemes are separated by dashed vertical lines. For clarity, the positions of the phoneme labels alternate up and down, with breath and silences (sil) shown in different colours.

In electropalatographic investigations of speech, a participant’s speech is recorded while wearing the artificial palate. Tongue contact with the palate activates the electrodes in the contact area, since these react electrically to the humidity of the saliva on the surface of the tongue. The speech recordings can consist of free speech, or more likely, a set of pre-selected sentences that are designed to investigate particular combinations of speech sounds. The temporal sequence of tongue–palate contact patterns is recorded in temporal registration with the acoustic signal. In the present study, the synchronised EPG and audio data were obtained from a freely available web database known as MOCHA (MultiCHannel Articulatory database: English) TIMIT [8], which consists of 460 English sentences that include the main connected speech processes and are read by female and male speakers. Figure 2 illustrates one sentence of the MOCHA TIMIT database; the soundwave (recorded at a 16 kHz sampling rate) is displayed with the corresponding phonemes separated by vertical dashed lines.

**Figure 3:**
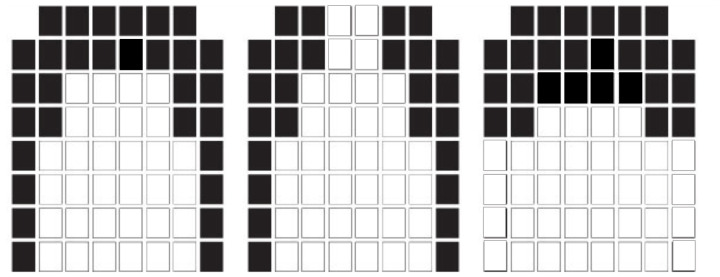
Illustration of different palatograms corresponding to different speech sounds, similar to [t] as in ’teen’, [s] as in ’seen’, and [l] as in ’lean’ (from left to right). Contact between electrodes and the tongue is indicated by black rectangles, while electrodes without contact are indicated by white rectangles. The top of the graph refers to the anterior part of the palate, the bottom the posterior part.

The synchronised EPG data are recorded in a separate file. Once a contact is registered by an electrode, an electrical signal is sent to an external processing unit [9], and a graphical display of the pattern of electrode excitation is shown either printed on paper or on a screen (Figure 3). When palatograms are shown on a screen, they can provide dynamic real-time visual feedback on the location and timing of tongue contacts with the hard palate. This direct articulatory information can be used during therapy to monitor and improve articulation patterns, especially in children [10,11,12]. Visual feedback is particularly important in rehabilitating children with hearing impairment. EPG has the potential to be useful in the assessment and remediation of a variety of speech disorders [13], including those due to hearing impairment [14], cleft palate [15], and Down’s syndrome [12]. The technique has also been used to study tongue–palate contact patterns for different inventories of vowels and consonants [16,17].

In phonetic studies of speech production, it has been implicitly assumed that the different postures for speech are symmetrical in the left–right plane of the vocal tract, i.e., that the contact between the tongue and the palate on the right-hand side is equally extensive as the contact on the left. Characterisation of articulation asymmetry in native speakers would contribute to a better understanding of the speech production process and its relationship with both neural organisation and the anatomy of speech organs. From a practical viewpoint, it could provide a reference for Speech and Language Therapists when treating speech deficiencies in which asymmetry plays a role (e.g., dysarthria). For example, if normative data show that a particular speech sound is often produced in a highly symmetrical way, then asymmetrical articulation of this speech sound resulting from weakness or paralysis of the muscles on one side of the mouth (which is very common in speech disorders due to stroke) could result in reduced intelligibility for the sound in question.

Asymmetries in tongue posture during the articulation of speech sounds have only been systematically investigated in a very small number of electropalatography studies (e.g., [18,19]). The general conclusion was that, irrespective of the language involved, the vast majority of palatograms show some degree of asymmetrical tongue–palate contact (i.e., there is at least one active electrode for which the electrode at the mirror position is inactive). There was, however, little consistency in the direction and amount of asymmetry, which seemed to differ depending on the individual speaker and/or the speech sound. Furthermore, these conclusions were based on the data of a very small number of speakers (i.e., a grand total of 15). A recent meta-analysis provides evidence from a much larger sample [20]. This study reviewed 1500 previously published palatograms representing a total of 225 speakers in 10 different languages. It was found that 83% of these palatograms showed some degree of asymmetrical contact. Palatograms with more tongue–palate contact on the left (45%) outnumbered those with more contact on the right (38%). The direction and amount of asymmetry depended on the place and manner of articulation. The results of this extensive review are being used to design an empirical EPG study with 20 speakers in which the direction and amount of asymmetry in tongue–palate contact will be studied as a function of (a) the type of speech sound, (b) anatomical asymmetries in speakers’ hard palates, and (c) speaker handedness. However, there may be a confounding factor due to the fact that the electropalate has been hand-made (a factor referred to herein as “palate manufacture”). Thus, even if there were no anatomical asymmetry, it could still be the case that electrodes in corresponding positions on the left and right side of the palate may not be positioned at equal vertical distances from the tongue. This could occur, for example, if the electropalate is not of perfectly uniform thickness such that electrodes placed on its surface do not exactly mimic the contours of the speaker’s hard palate. It could also occur if two corresponding electrodes (one on the left and one on the right) are not positioned at equal distances from the mid-sagittal plane. In summary, asymmetry in the palatogram for a given speech sound may arise from three sources: the shape of the speaker’s hard palate (i.e., anatomy), the positioning of the electrodes (i.e., palate manufacture) and the movement of the tongue.

The present paper describes a series of tools for (i) the analysis of asymmetry in palatograms and (ii) the distinction between asymmetry that arises from anatomical or manufacturing causes and asymmetry that is due to the movement of the tongue. Firstly, it introduces processing algorithms that allow the analysis of asymmetry in palatograms and that enable asymmetry metrics to be calculated for different speech sounds. Secondly, it describes an image processing framework that segments the main elements of an EPG palate to analyse the locations of the electrodes with respect to the sagittal plane. This provides information about asymmetry that arises due to a combination of speaker anatomy and palate manufacture.

A preliminary version of this work, presented at the 22nd Medical Image Understanding and Analysis (MIUA) Conference [21], focused solely on the imaging and segmentation of the artificial palate. The present study also describes the analysis and visualisation of speech signals, and the derivation of asymmetry metrics from palatograms. Furthermore, the algorithms have been made publicly available through GitHub (see Supplementary Materials).

## 2. Display and Analysis of Audio Signals and Palatograms

Whilst there are useful software packages to visualise and analyse speech (e.g., *Praat* [22]), researchers sometimes require more flexibility to analyse data of interest. Thus, this section describes a series of MATLAB^®^ (The Mathworks™, Natick, MA, USA) routines that are available open-source in the *GitHub* software development platform [23]. The most important functions are contained in the main folder (https://github.com/reyesaldasoro/ElectroPalatography). The extension .m indicates a file in which a MATLAB^®^ function is saved, e.g., interpretLabelledPhonemes.m. Alternatively, the functions can be presented as they are used with input and output parameters, e.g., output = function(input); for example: TextGrid = Lab_to_TextGrid(dataIn);.

### 2.1. Conversion between Data Formats

The MOCHA TIMIT database, in addition to containing synchronised EPG and acoustic data, has a .lab (‘label’) file saved for each sentence produced by each speaker. In this file, each phoneme is represented by a row, which contains the start and finish times of the phoneme followed by a code to identify the phoneme. An example of the first 8 lines of the .lab file for the sentence ”This was easy for us” is shown in the Appendix A.

In phonetics research, a common means of recording the start and finish times of phonemes is via a TextGrid file, a proprietary format of the popular software Praat. These files have a more complicated format than the MOCHA TIMIT .lab files, with markup fields that allow words and phonemes to be identified. The files use the extension .TextGrid and an example of the first part of a file is shown in the Appendix A.

Conversion from .lab to .TextGrid could be a valuable tool for researchers who wish to use the MOCHA TIMIT database. It is performed with the function TextGrid = Lab_to_TextGrid(dataIn);. The input parameter dataIn can be either a file name (which is read, converted to .TextGrid and saved in the folder where the .lab file is located) or a folder, in which case all the .lab files in the folder are converted to .TextGrid.

The code requires one intermediate function to convert the .lab file to a MATLAB^®^ cell. This function can be used separately to analyse, for example, acoustic or EPG data at the level of the phoneme in MATLAB^®^. There is a parallel function to read a .TextGrid file and convert it to a MATLAB^®^ cell. Both of these functions are called from the function interpretLabelledPhonemes.m, which reads a file name as the input and, as the output, produces a MATLAB^®^ cell containing the words (if available) and the phonemes along with their corresponding start and finish times. The function automatically detects the name of the file (it can end in ”d” for .TextGrid or ”b” for .lab), and calls the correct function, either convert_LAB_to_Phonemes.m or convert_TextGrid_to_Phonemes.m.

### 2.2. Sound Waves and Palatograms

In the MOCHA TIMIT database, the sound waves are saved as audio files, with the extension .wav, and the palatograms are saved in a file with the extension .epg.

The function readAudioFile.m reads the audio file and automatically calculates some important parameters that will be used later on: sample rate, number of samples, maximum and minimum amplitudes.

The process of reading the electropalatography data from an .epg file requires several files: readPalatogram.m, EPG_to_Palatogram.m, asymmetry_projection.m and EPG_Boxes.mat. These files are necessary as many parameters are calculated in this step, including the whole time sequence of palatograms and their asymmetry characteristics (to be described below). It is recommended to read the audio file prior to reading the EPG data, as the audio sample rate is necessary for some calculations.

### 2.3. Spectrum Through a Short-Time Fourier Transform

**Figure 4:**
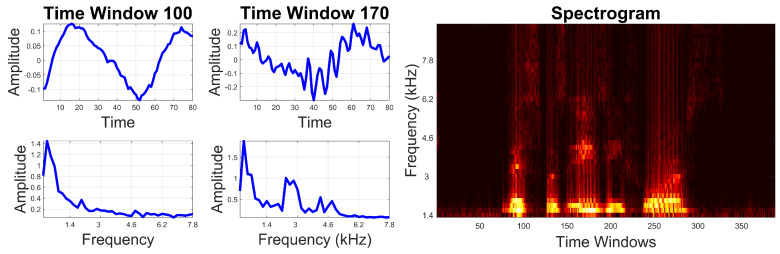
Illustration of a signal in the time and Fourier domains. A sound wave, such as the one shown in Figure 2, can be partitioned into small windows (top left) and then transformed into the Fourier or frequency domain (bottom left) where the frequency components of the sound are displayed. Note that the sound signal displayed in window 100 is a low-frequency signal that closely resembles a sinusoid, whilst the sound signal displayed in window 170 has both low- and high-frequency components (a base sinusoid with fast, noise-like variations). The transforms for consecutive time windows can be placed together on a spectrogram with time on the horizontal axis, frequency on the vertical axis and amplitude encoded as brightness.

The Fourier Transform [24], which is often calculated using the popular Fast Fourier Algorithm [25], is a well-known mathematical operation that translates a signal from the spatial or time domain into the Fourier or frequency domain. The transformation of sound signals into the Fourier domain is widely used in phonetics, as it provides information about how the energy contained in each phoneme is distributed along the frequency spectrum. Different calculation methods are possible, which lend themselves to different display formats (see Figure 4). For example, if the transformation is applied to a time window corresponding to an entire phoneme, the frequency components of the phoneme can be visualised by showing the amplitude of the Fourier signal on the vertical axis as a function of the frequency on the horizontal axis. Alternatively, an audio signal can be partitioned into smaller time windows, each of which is transformed separately. The amplitude is then converted to brightness and arranged vertically, where the vertical axis represents frequency. Columns of brightness corresponding to consecutive time-points (or window numbers) are then arranged sequentially (from left to right) to form an image known as a spectrogram. Thus, the level of brightness at any point in the image represents the amplitude of the Fourier signal at a particular frequency and time. The short-time Fourier Transform of the audio signal is calculated with the function shortTimeFourierAnalysis.m, which is used in the following way: EPG_parameters=shortTimeFourierAnalysis(EPG_parameters);.

### 2.4. Asymmetry Visualisation

**Figure 5:**
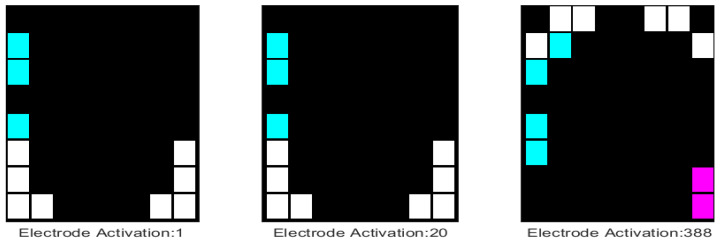
Illustration of asymmetry in individual palatograms corresponding to different time points. Asymmetry is indicated with colours; cyan (pink) shows electrodes activated on only the left (right) side of the palate, while the white electrodes are activated on both sides of the palate. The top of the graph refers to the anterior part of the palate, the bottom the posterior part.

**Figure 6:**
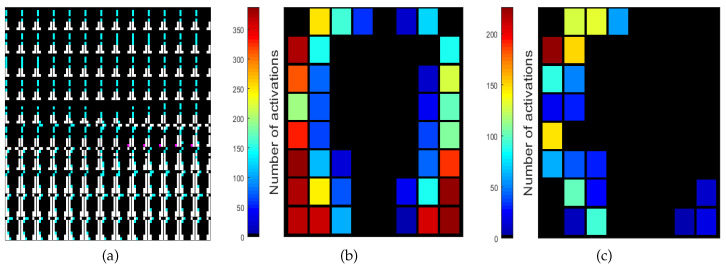
Illustration of different methods of highlighting asymmetry. (**a**) A montage of 130 palatograms in which the asymmetry is indicated with colours; cyan/pink shows electrodes activated on only one side of the palate; (**b**) Cumulative electrode activation in which the count increases every time an electrode is activated and colours in the bar correspond to the number of activations; (**c**) Cumulative asymmetric activation in which activity is only recorded when the electrode is activated asymmetrically, that is, without its corresponding mirror electrode.

Asymmetry in an EPG investigation refers to the outcome where electrodes are activated on one side of the palatogram, whilst those in the mirror position are not. The software described here allows the analysis of asymmetry in several ways. First of all, each palatogram is analysed for asymmetry; electrodes are recorded as either active in pairs (symmetric) or active on the left/right side only (Figure 5). The information per palatogram is saved within one of the fields of EPG_parameters, which is a structure. Specifically, it is saved in EPG_parameters.PalatogramAsym(:,:,:,1), where 1 indicates that the first palatogram in the sequence is the palatogram of interest. Thus, asymmetry maps for individual palatograms can be displayed with the following command: imagesc(EPG_parameters.PalatogramAsym(:,:,:,1)). Montages are a useful means of visualising asymmetry maps for a sequence of palatograms simultaneously, which can be achieved using the following command: montage(EPG_parameters.PalatogramAsym(:,:,:,1:130)). An example of a montage is shown in Figure 6a.

Two types of cumulative image can be generated to display the pattern of asymmetry across a sequence of images. First, the activation of each electrode is accumulated to provide a map that shows the total activation of the electrodes. Such an image can be used to observe whether the activation is symmetrical on the left and right side of the palate and can be displayed in the following way: displayPalatogram(EPG_parameters,-1) (Figure 6b). Second, the activation of the electrode is accumulated only if it was activated asymmetrically, i.e., only if the corresponding electrode in the mirror position was not activated. This can be displayed in the following way: displayPalatogram(EPG_parameters,-2) (Figure 6c). Both of these display formats allow the pattern of asymmetry across a sentence to be observed in a single image.

**Figure 7:**
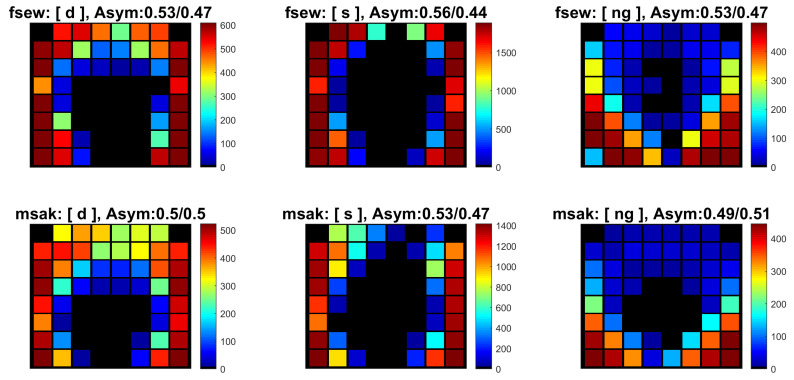
Visualisation of asymmetry on a per-phoneme basis. All the occurrences of three selected phonemes, [d, s, ng], were extracted from 69 sentences for two speakers (*fsew, msak*) of the MOCHA TIMIT data base. The accumulation of the activation of each electrode is reflected by the colour. The asymmetry values for the cumulative palatograms are shown in the titles.

The previous visualisations are useful as an indication of the average asymmetry for a given sentence or speaker. However, as mentioned in Section 1, the direction and amount of asymmetry are likely to depend on the type of speech sound. Thus, it could be of interest to analyse the pattern of asymmetry as a function of individual phonemes. The specific tools for a per-phoneme approach are described in the folder ElectroPalatography/extractPhonemes/, and can be invoked by running the file ComparePhonemes.m. The user selects a set of phonemes of interest, for instance:  listPhonemes = {’d’,’s’,’ng’}; (where ’ng’ is the phoneme that occurs at the end of the word ’sing’, for example), as well as a folder containing the set of sentences to be analysed. For each occurrence of each phoneme in this set of sentences, the programme extracts a cumulative palatogram that is obtained by summing over all the palatograms corresponding to the duration of the phoneme. These palatograms are stored as a 3D matrix in column 2 of the variable avPhoneme_tot. The following illustrations were obtained from 69 different sentences of the MOCHA TIMIT database (chosen at random), uttered by 2 different speakers called *fsew* and *msak*. The first letter *f/m* corresponds to female/male and the remaining three letters correspond to the initials of the speaker. The asymmetry pattern for these two speakers for the three phonemes mentioned above is shown in Figure 7. Note that these palatograms indicate the accumulation of the individual electrode activations over time across (a) all occurrences of the phoneme within the set of 69 sentences and (b) the total duration of each occurrence of each phoneme. It can be seen that speaker *fsew* exhibits greater asymmetry than speaker *msak*.

In addition to the visualisation, metrics representing the amount and direction of asymmetry in the palatogram are calculated in two different ways: (a) The number of activations on the left/right side of the palatogram is divided by the total number of activations. Thus, a perfectly symmetrical case would have a result 0.5/0.5, whilst a perfectly asymmetrical case would correspond to either 1/0 or 0/1 (note that the sum of the two values is always equal to 1). The two values are stored in columns 3 and 4, respectively, of the variable avPhoneme_tot. The asymmetry values reported in Figure 7 use this notation. (b) A single asymmetry metric is defined as: (number of right activations - number of left activations) / (number of right activations + number of left activations). Thus, a perfectly symmetrical case would have a result 0, whilst a perfectly asymmetrical case would correspond to either +1 or −1. These values are stored for each phoneme occurrence in column 9 of avPhoneme_tot.

**Figure 8:**
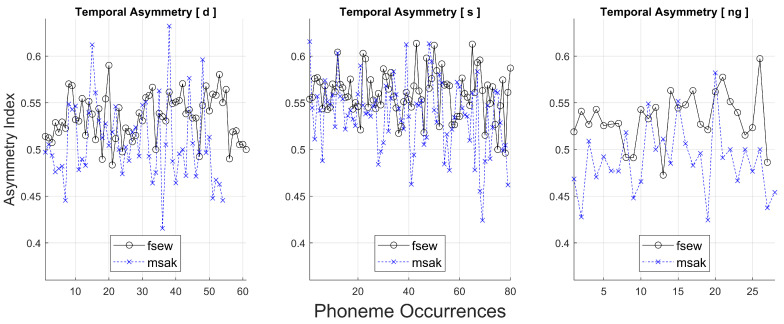
Variation of the asymmetry of three selected phonemes, [d, s, ng], for two speakers (*fsew, msak*). Each data point corresponds to a different realisation of the phoneme in a set of 69 sentences. Note that for all phonemes, the average asymmetry corresponding to speaker *fsew* is higher than that of speaker *msak*. For both speakers, the phoneme [s] exhibits the greatest amount of asymmetry.

It is also possible to visualise how the asymmetry metric varies across different realisations of the same phoneme. This is illustrated in Figure 8. Thus, for the phoneme [s], for example, there were approximately 80 occurrences of the phoneme across the 69 sentences. The asymmetry index shown on the y-axis was obtained according to method (a) described above, where only the first value is shown (column 3 of the variable avPhoneme_tot). In phonetics, the term ”coarticulation” is used to refer to the fact that in continuous speech, the articulatory features of an individual speech sound may be influenced by the phonetic context, i.e., the preceding and/or subsequent speech sound. The MOCHA TIMIT dataset was designed to test a wide variety of the phoneme sequences that occur in English. Thus, the variation in asymmetry across different realisations of the same phoneme in Figure 8 is mainly due to variation in the adjacent speech sounds. In [26], we use the MOCHA TIMIT data to show that [r] and [l] exhibit greater variability in asymmetry than other phonemes, which is in line with literature suggesting that these sounds are particularly prone to coarticulatory effects. Future work will also examine the change in the pattern of asymmetry across the duration of individual phonemes, for different phonemes produced in a variety of phonetic contexts.

## 3. Segmentation and Analysis of CT Images of the Electropalate and Plaster Cast

**Figure 9:**
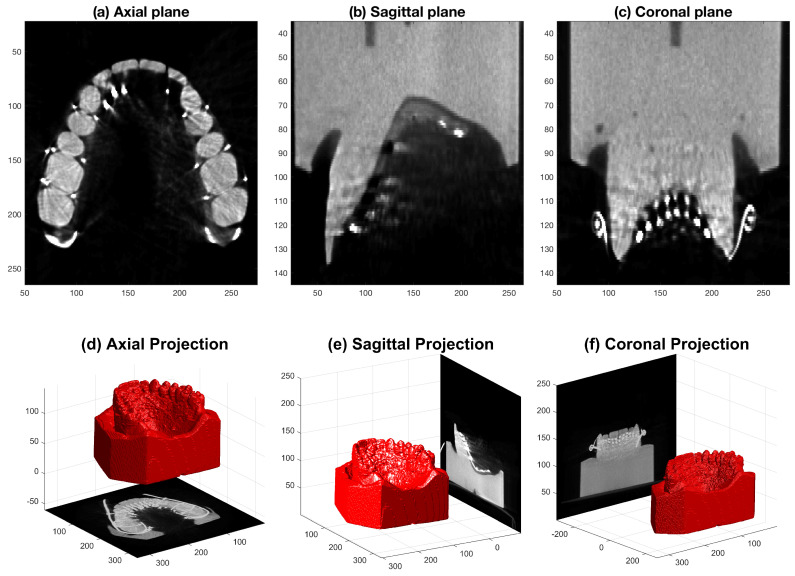
Representative slices of a CT scan of an EPG palate fixed in place on the corresponding plaster cast. (**a**) Axial slice showing the tip of the teeth with the molars at the bottom of the image and the incisors at the top. (**b**) Sagittal slice with the frontal teeth at the lower left. (**c**) Coronal slice showing the cusps of the canines. The images in the second row (**d**–**f**) illustrate the positions of these projections in 3D space, as they are shown with a 3D rendering of the cast. The circular lines in (**c**) correspond to the metallic clips that hold the electrical cables. Note that although there are differences in intensity between the cast of the palate (on the one hand), and the electrodes and wires (on the other), there are some regions of the cast that are as bright as the electrodes. These are especially noticeable in the lower molars in the axial plane and in the canines in the coronal plane. See Figure 10, where the regions have been incorrectly segmented by intensity alone.

In this section, a method is presented in which computed tomography (CT) images of the artificial palate and plaster cast are analysed in order to quantify the asymmetry that arises from a combination of anatomical and manufacturing causes. Images of the stone cast and the palate, shown in Figure 1, were acquired with a SCANORA^®^ 3Dx Cone Beam CT system by Soredex (KaVo, Biberach, Germany). Two hundred and fifty DICOM^®^ axial images of resolution 333×333 pixels with pixel and slice spacing of 0.3 mm at a power of 85 kiloVolts peak (kVp) were acquired (Figure 9). The images of the palate and the cast showed a difference in intensity between the background (black), the cast (dark grey) and the metallic elements (light grey to white). However, the metallic elements created some artefacts elsewhere in the image, such as the streak lines that can be seen in the molars of Figure 9a, which complicated the segmentation.

### 3.1. Intensity Segmentation

**Figure 10:**
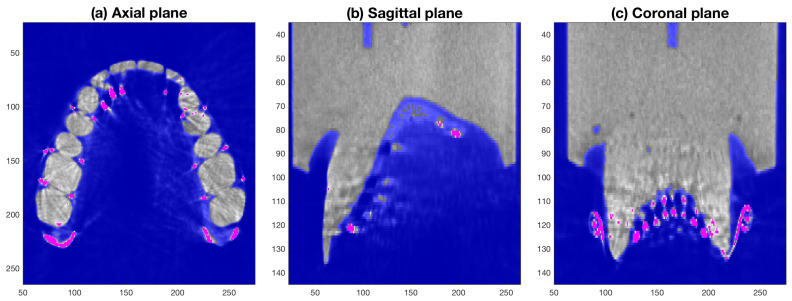
Segmentation with double thresholding. The images of Figure 9 were segmented with a low threshold to segment the background (blue shade) and a high threshold to segment the metallic elements (pink shade). Note that the high threshold does not select all the electrodes in (**b**), nor does it discard high intensity pixels of the cast clearly visible in (**a**,**c**).

Since the metallic elements of the electrodes and wires are denser than the cast of the palate, it is possible to exploit the corresponding difference in intensity on the CT images. However, the material of the cast of the palate is not perfectly uniform and the artefacts previously mentioned did not allow segmentation by a single threshold. Thus, a segmentation involving two intensity thresholds [27], inspired by the Schmitt trigger [28], was followed: a lower intensity threshold to segment the background (dark pixels) and a higher intensity threshold to segment the metallic elements (bright pixels). The background was easily segmented with a low threshold; however, a high threshold was not sufficient to adequately segment the metallic elements, as some voxels of the cast displayed a very high intensity and were confused as metal (Figure 10).

**Figure 11:**
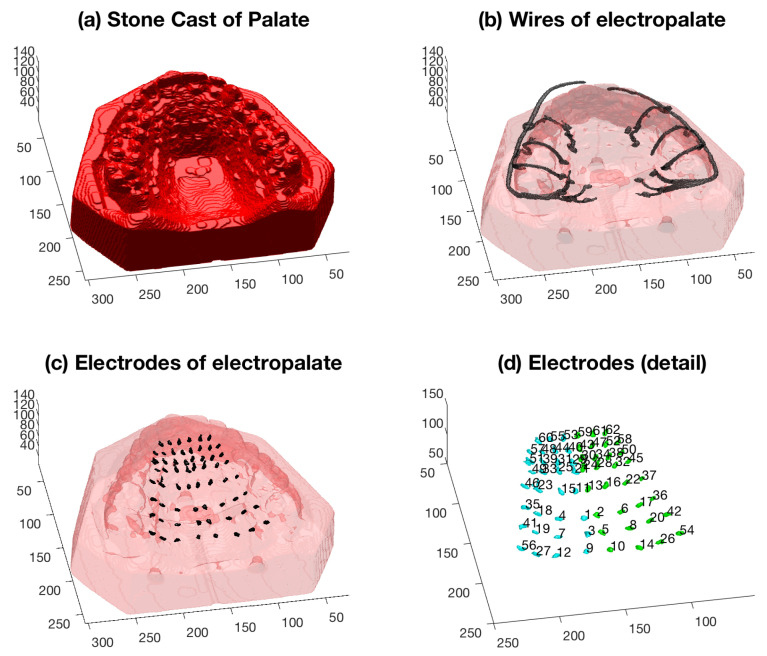
Segmentation of the structures of interest. (**a**) Segmentation of the cast of the speaker’s hard palate; (**b**) Segmentation of the wires that hold the electropalate in place; note the detail that is captured by the segmentation in the small loops that go through the teeth. The segmentation of the cast is shown with transparency for reference; (**c**) Segmentation of the electrodes; (**d**) Electrodes shown in more detail; the electrodes have been uniquely labelled and grouped into left (green) and right (cyan) sides. Note that the palate corresponds to the upper jaw and therefore is upside down.

The higher threshold was further divided into two new high thresholds, which roughly corresponded to the wires that held the palate in place, and the electrodes and their connections. The segmentation of the stone cast was refined by using a series of morphological operations: closing, opening and filling of holes (Figure 11a). This allowed a solid region to be determined and subsequently all high intensity voxels detected in that region were discarded. The wires (Figure 11b) were morphologically segmented from the electrodes (Figure 11c) by size. Once the electrodes were segmented from the cast and the wires, these were uniquely labelled (Figure 11d).

### 3.2. Plane of Symmetry

**Figure 12:**
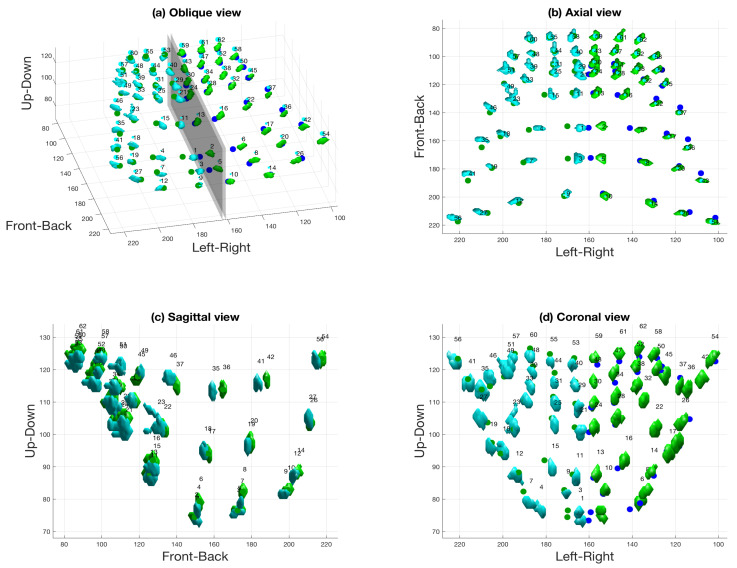
(**a**) Illustration of the plane of symmetry used to group electrodes into left and right sides. (**b**) Axial view. (**c**) Sagittal view. (**d**) Coronal view. In all cases, left electrodes are shown in green and right electrodes are shown in cyan. The green and blue dots denote electrodes that have been projected to the opposite side to illustrate the asymmetry in electrode positioning.

Since the cast had been correctly oriented during the acquisition of the CT, the plane of symmetry was considered as a sagittal plane in the front-back direction and was located in the gap between the two frontal teeth. This plane was used to divide the electrodes into the left and right sides of the palate (Figure 12a).

### 3.3. Projection of the Electrodes and Calculation of Asymmetry

The final step of the algorithm consisted of a projection of each electrode through the plane of symmetry to the opposite side of the palate. If the speaker’s hard palate had been perfectly symmetrical and the electrodes had been perfectly located, the projection should land exactly over the corresponding opposite electrode. Figure 12b,c,d show the electrodes and their corresponding projections (the latter are shown as green and blue dots) in the three views.

**Figure 13:**
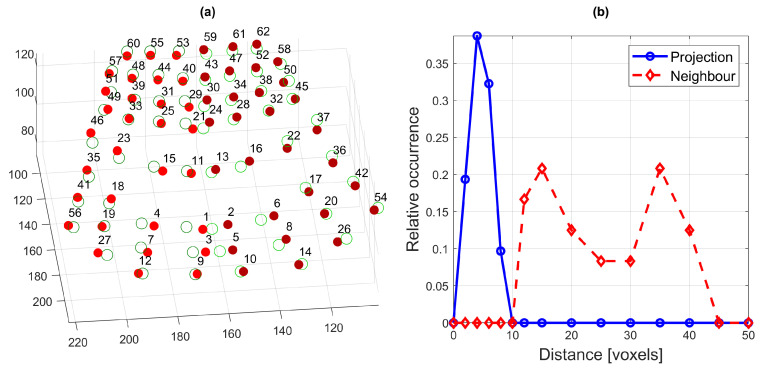
Analysis of the asymmetry of the artificial palate: (**a**) The positions of the electrodes (filled spheres) and their projections over the plane of symmetry (hollow spheres); (**b**) Histograms of the distance between electrodes and their mirror-image projections (solid blue line) and between neighbouring electrodes (red dashed line).

Finally, the following two distance metrics were calculated (see Figure 13): (a) the distance between each electrode and the projection of its mirror-image counterpart (e.g., 9 and 10), and (b) the distance between two neighbouring electrodes (e.g., 9 and 12). In both cases, the distance refers to the magnitude of the 3D vector between the two electrodes. The average distance between electrodes and their reflections, in units of voxels, was 4.5±1.8 (mean ± one standard deviation), with a minimum of 1.2 (between electrodes 32 and 33) and a maximum of 8.5 (between electrodes 4 and 6). On the other hand, the mean distance between neighbouring electrodes was 24.3±10.15 voxels, with a minimum of 11.38 (between electrodes 40 and 44) and a maximum of 42.27 (between electrodes 12 and 27). Figure 13 illustrates these results by showing (a) the electrodes and the projections of their mirror-image counterparts and (b) histograms of the two distance metrics described above. As can be seen, the distances between neighbouring electrodes are much larger than the distances between electrodes and their reflections (in fact, the distributions do not overlap at all). Thus, it is possible to conclude that, for this speaker, any asymmetry observed in the speaker’s palatograms would be due to asymmetrical movement of the tongue rather than due to the anatomy of the hard palate or the manufacture of the electropalate.

All the image processing was performed in Matlab^®^ (The Mathworks^TM^, Natick, MA, USA) and the code is available upon request.

## 4. Discussion

The segmentation steps described above provided a successful segmentation of the electrodes as single elements that were uniquely located in the three-dimensional space. In the case of this particular palate, any asymmetry observed in a palatogram is due only to the positioning of the tongue and not due to the shape of the hard palate of the speaker and/or irregularities in the palate manufacture.

From Figure 12b, it can be seen that the majority of the electrodes corresponded to their mirror position, especially in the anterior section and the bottom row of the palate; that is, the dots are barely visible, as they coincide with the positions of the electrodes. A few electrodes seem to be displaced in the front-back direction (electrodes 35, 36, 41, 42), while others are displaced in the left–right direction (electrodes 1, 2, 3, 5). The displacement in the up-down direction, which is the main direction of interest, can be appreciated in Figure 12c,d. It can be observed that the displacement is very small, as there are no dots to be seen in the sagittal view, and in the coronal view, the visible dots are mainly due to the left–right displacement seen in the axial view. In the case of this particular palate, any asymmetry observed in a palatogram is due only to the positioning of the tongue and not due to the shape of the speaker’s hard palate and/or irregularities in the palate manufacture.

This work provides a set of tools that can be used in the analysis of electropalatography, and especially in the analysis of asymmetry – both asymmetry in the pattern of activation recorded by the electropalate, and asymmetry in electrode positioning due to the speaker’s anatomy and/or palate manufacture. There is considerable interest in the asymmetry of the mandible [29,30] and the shape and morphometry of the palatal rugae [31,32]. As mentioned in Section 1, future work will aim to determine whether speaker handedness influences left–right asymmetry in tongue movement. To this end, individuals will be selected in two groups, each with different hand-dominance.

Another future direction will be the creation of three-dimensional visualisation outputs, which better reflect the anatomy of the phenomenon than the palatograms previously shown. This may be particularly useful in therapy where the provision of real-time visual feedback of tongue movement can be effective in the remediation of certain types of intractable speech problem.

Finally, this paper has established the methodology for producing *master calibration* palatograms for different speech sounds. These palatograms could capture the most prototypical realisation of the speech sounds of English provided that a large enough sample would be available. The processing tools presented in this paper allow determination of the variability in the pattern of tongue–palate contact due to individual variation and varying phonetic context. Such information could be used to objectively assess speech accuracy. It should be kept in mind, however, that the relationship between speech production and speech perception may be more complex than suggested here, especially in clinical populations.

## Supplementary Materials

The code associated with this article is available at https://github.com/reyesaldasoro/ElectroPalatography.

## Appendix A. Examples of File Formats

An example of the first 8 lines of the .lab file for the sentence ”This was easy for us”:

0 0.25 sil
0.25 0.28 breath
0.28 0.35 sil
0.35 0.42 dh
0.42 0.5 i
0.5 0.61 s
0.61 0.64 w
0.64 0.68 @

An example of the first part of a .TextGrid file:

File type = ”ooTextFile”
Object class = ”TextGrid”

xmin = 0
xmax = 3.968
tiers? exists
size = 1 item []:
     item [1]:
              class = ”IntervalTier”
              name = ”phonemes”
              xmin = 0
              xmax = 3.968
              intervals: size = 51
              intervals [1]:
                   xmin = 0
                   xmax = 0.7904913168586506
                   text = ””
                   intervals [2]:
                   xmin = 0.7904913168586506
                   xmax = 0.8708421929714597
                   text = ”g”

Example of the reading and displaying of the start and finish times of the phonemes that comprise the first sentence of the MOCHA TIMIT database (”This was easy for us”). In this example, the sentence was uttered by one of the female speakers (with initials “sew”):

>> currentLAB_File = ’MOCHA/fsew0_v1.1/fsew0_001.lab’;
>> [EPG_parameters] = interpretLabelledPhonemes(currentLAB_File);
>> disp(EPG_parameters)
            LAB: {18 x 3 cell}
    numPhonemes: 18
          Words: []
       numWords: 0

>> disp(EPG_parameters.LAB)
    [     0]    [0.2500]    ’sil’
    [0.2500]    [0.2800]    ’breath’
    [0.2800]    [0.3500]    ’sil’
    [0.3500]    [0.4200]    ’dh’
    [0.4200]    [0.5000]    ’i’
    [0.5000]    [0.6100]    ’s’
    [0.6100]    [0.6400]    ’w’
    [0.6400]    [0.6800]    ’@’
    [0.6800]    [0.7500]    ’z’
    [0.7500]    [0.9200]    ’ii’
    [0.9200]    [0.9800]    ’z’
    [0.9800]    [1.1100]    ’iy’
    [1.1100]    [1.1800]    ’f’
    [1.1800]    [1.2500]    ’@’
    [1.2500]    [1.3500]    ’r’
    [1.3500]    [1.4200]    ’uh’
    [1.4200]    [1.6700]    ’s’
    [1.6700]    [2.2000]    ’sil’
